# Infliximab Combined with Enteral Nutrition for Managing Crohn's Disease Complicated with Intestinal Fistulas

**DOI:** 10.1155/2016/5947926

**Published:** 2016-09-22

**Authors:** Xiao-Li Wu, Ren-Pin Chen, Li-Ping Tao, Jian-Sheng Wu, Xiang-Rong Chen, Wei-Chang Chen

**Affiliations:** ^1^Department of Gastroenterology, First Affiliated Hospital of Soochow University, Suzhou 215000, China; ^2^Department of Gastroenterology, First Affiliated Hospital of Wenzhou Medical College, Wenzhou, Zhejiang Province 325000, China

## Abstract

*Aim*. This study was performed to evaluate the additional enteral nutrition (EN) in the efficacy of infliximab (IFX) compared with the conventional therapy in managing Crohn's disease (CD) complicated with intestinal fistulas.* Methods*. A total of 42 CD with intestinal fistulas were randomly divided into infliximab treatment group (*n* = 20) and conventional therapy group (*n* = 22). We evaluated the laboratory indexes, Crohn's disease activity index (CDAI), Crohn's disease simplified endoscopic score (SES-CD), and healing of fistula in the two groups before treatment, at 14 weeks, and at 30 weeks, respectively.* Results*. In the IFX treatment group, the CDAI score, the SES-CD, erythrocyte sedimentation rate, and C-reactive protein levels were significantly decreased during treatment compared with those before treatment. The body mass index and albumin levels were increased in both groups. Moreover, in the IFX treatment group, fistula healing was found in 8 at the 14th week and 18 at the 30th week, respectively, which was greater than that in the conventional therapy group.* Conclusion*. Our study suggested that infliximab combined with EN is an effective treatment for CD patients complicated with intestinal fistulas.

## 1. Introduction

Crohn's disease (CD) is a chronic granulomatous disorder characterized by the presence of inflammatory ulcerative lesions in the gastrointestinal tract. Although CD has been traditionally defined as a chronic inflammatory condition that can be located in any part of the gastrointestinal tract, most sites located at the terminal ileum and the proximal colon [1]. In the last two decades, the incidence of CD has been gradually increasing in Asian countries including China, Japan, and South Korea [2]. CD can easily be complicated by the formation of intestinal fistulas. To date, treatment of CD has been geared towards symptomatic relief of disease exacerbations via pharmacological interventions such as aminosalicylates, corticosteroids, immunosuppressive agents, antibiotics, and nutritional therapy. However, there are still around 50% of patients who have failed to respond to medical management that need surgical interventions to correct intestinal obstruction or abscesses. Moreover, surgical intervention is curative for ≥80% of patients with recurring after intestinal resection. Infliximab (IFX) is a murine chimeric monoclonal antibody direct against human tumor necrosis factor- (TNF-) alpha [3]. For moderate-to-severe CD and fistulizing CD, usage of IFX in early stage can effectively induce remission, promote the healing of fistula, and prevent recurrence [4]. Most of the patients who have CD with intestinal fistula had a high prevalence of malnutrition. Clinical application of enteral nutrition (EN) generally can improve the nutritional status of patients with CD and promote the remission of the disease. Thus, in this study, we investigated the potential application of EN in CD patients with intestinal fistulas undergoing infliximab treatment.

## 2. Materials and Methods

### 2.1. Patients

Patients (*n* = 42) who had CD complicated with intestinal fistulas, admitted between December 2012 and September 2015 in the First Affiliated Hospital of Wenzhou Medical University, Zhejiang Province, Southeast China, were recruited for this study. The cases were randomly divided into infliximab treatment group (*n* = 20) and conventional therapy group (*n* = 22) by the table of random number. This study was approved by the hospital ethics committee. Oral informed consents have been obtained from patients. Eligible patients were men and women who were (1) 18 years of age or older, (2) diagnosed according to the 2012 “China Diagnostic Criteria for the Diagnosis of Inflammatory Bowel Disease [5] Standard Treatment Consensus,” (3) diagnosed, classified, and estimated as fistula according to the American Gastroenterological Association (AGA) [6], and (4) under the clinical criteria with an indication for IFX treatment. Patients who received less than six IFX injections or had infection diseases (such as TB, HIV, and viral hepatitis), heart disease or diabetes, and short bowel syndrome were excluded in this study.

### 2.2. Treatment

Twenty-two patients in the conventional treatment group were treated with methylprednisolone and azathioprine. For patients whose symptoms were in remission in the conventional treatment group, usage of methylprednisolone was decreased by 5 mg every week until 20 mg per day, and then the amount was decreased by 2.5 mg every week. Azathioprine (Imuran: 1.5–2.5 mg/kg/day) was fully given for 32 weeks. Among these patients, 5 were treated with methylprednisolone and azathioprine two weeks after the surgery.

For the IFX therapy group, 20 patients received a 32-week intravenous injection of 5 mg/kg at 0, 2, and 6 weeks followed by once every 8 weeks. Among these patients, 6 were treated with IFX two weeks after the surgery.

#### 2.2.1. Surgical Drainage

A total of 11 patients with “complex” fistula, 6 from IFX group and 5 from the conventional therapy group, were treated with incision and drainage of abscess, side-to-side anastomosis, and antibiotics.

#### 2.2.2. Enteral Nutrition

Patients (*n* = 42) received EN (a mixed suspension from Peptison liquid, Nutricia Pharmaceutical Co., Ltd.) by nasogastric tube (Huarui Pharmaceutical Company) every night for 2 months. Approximately 1000 mL of EN formula (1000 kcal/night) was given with a droplet velocity of 80–120 mL/h.

### 2.3. Evaluation of Outcomes

#### 2.3.1. Laboratory Indicators

Laboratory examination results, including blood routine test, aspartate aminotransferase (AST), alanine aminotransferase (ALT), body mass index (BMI), albumin (ALB), erythrocyte sedimentation rate (ESR), and C-reactive protein (CRP), were collected before treatment, on the 1st day, and at 14th and 30th week, respectively. Patients' symptoms, including abdominal pain, diarrhea, bloody stool, perianal lesions, and extraintestinal manifestations, were also recorded.

#### 2.3.2. CDAI

The CDAI score was estimated according to the “China Inflammatory Bowel Disease Diagnosis and Treatment Methods of Normative Consensus” [5].

#### 2.3.3. Endoscopy Examination

Colonoscopy or double-balloon endoscopy was performed before treatment (simple endoscopic score for CD [SES-CD] [7]) and at the 14th and 30th weeks, respectively.

#### 2.3.4. Fistula Healing

The evaluation criteria of the Sands [8] treatment for fistula were as follows: (1) complete response: fistula had disappeared; (2) partial response: size was reduced or the opening of the fistula drainage was smaller; and (3) no response: the disease was still active and the fistula drainage showed no obvious improvement.

#### 2.3.5. Abdominal MRI Examination

Abdominal MRI examination was performed before and after 30-week treatment.

*Adverse Effects*. Any adverse events occurred during the treatment were recorded.

### 2.4. Statistical Analysis

The data were analyzed using the SPSS 17.0 statistical software (SPSS, IL, USA). The Wilcoxon signed rank-sum test was used for analysis. Count data was analyzed using Fisher exact test or *χ* ^2^ test. A *P* value of <0.05 was statistically significant.

## 3. Results

### 3.1. Characteristics of the Patients

Forty-two CD patients with intestinal fistulas who were diagnosed by clinical, endoscopic, histopathological, and radiological examinations were evaluated (shown in Table 1). The major duration of disease (*n* = 27, 64%) was 12–36 months. Among these patients, 12 were ileum type, 13 were colon type, and 17 were ileum + colon type. All of them were penetration type. Twenty-seven of patients (64%) were at moderate active stage, while 15 were at severe active stage. A total of 11 patients had intestinal surgery history, and 8 had extraintestinal manifestations. The clinical features such as age and gender were not significant in the two groups (*P* > 0.05).

### 3.2. Evaluation of Effects

#### 3.2.1. Laboratory Data

The laboratory examination data and BMI of the two groups are shown in Table 2.

In the IFX treatment group, compared to the level before treatment, the CRP and ESR levels were significantly decreased at 14 weeks and 30 weeks (all *P* < 0.05), while ALB increased after 14 weeks and 30 weeks (all *P* < 0.05). The BMI increased from (17.52 ± 1.89) kg/m^2^ to (19.19 ± 2.13) kg/m^2^ at 14 weeks (*P* < 0.05) and to (20.26 ± 2.65) kg/m^2^ at 30 weeks (*P* < 0.05). In the conventional group, the CRP and ESR levels were decreased, too. The BMI increased from 17.66 ± 1.98 kg/m^2^ to 18.21 ± 2.09 kg/m^2^ at 14 weeks and to 19.98 ± 2.49 kg/m^2^ at 30 weeks in the conventional group. However, the difference between the two group was not statistically significant. Moreover, in the two groups, ALT and AST were not significantly changed before and after treatment.

#### 3.2.2. Clinical Manifestations

According to the answers of questionnaires from patients, syndromes of abdominal pain, diarrhea, and hematochezia were relieved in the IFX treatment group (*n* = 19) and in the conventional therapy group (*n* = 16).

**Figure 1:**
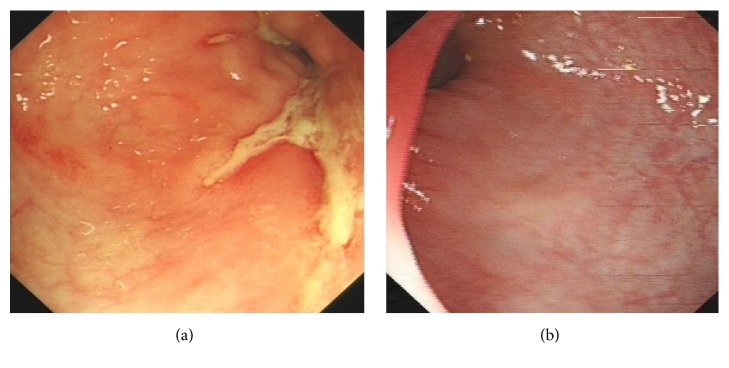
(a) Ulcer in the sigmoid colon before treatment of one case in the IFX treatment group; (b) sigmoid colon 30 weeks after treatment (no ulcer is seen).

In the IFX treatment group, after 14 weeks of treatment, CDAI score was significantly decreased compared to that before treatment (*P* < 0.05). The SES-CD decreased from 8.6 ± 2.2 to 3.5 ± 0.8, and the difference was statistically significant (*P* < 0.05). After 30 weeks of treatment, the CDAI score decreased to 125.6 ± 42.5, which was statistically significant (*P* < 0.05) compared to that before treatment. And the SES-CD significantly decreased (*P* < 0.05). Compared to those in the conventional therapy group, the difference of CDAI and SES-CD between the two groups was significant (*P* < 0.05) (Figure 1).

**Figure 2:**
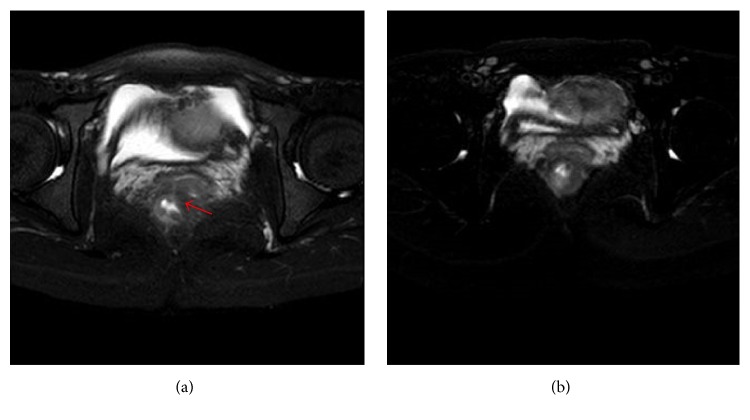
(a) Rectovaginal fistula before treatment (red arrow) of one case in the IFX treatment group; (b) rectovaginal fistula cured after the sixth course of IFX treatment.

Fistula closure was found in 8 patients at 14 weeks and in 18 at 30 weeks, respectively. Moreover, one patient with rectovaginal fistula was healed after the sixth course of IFX treatment (Figure 2). The cases of fistula healing in IFX treatment group were higher than that in conventional therapy group at 30 weeks (*P* < 0.05) (Table 3).

#### 3.2.3. Adverse Effects

Of the 20 patients in IFX treatment group, 15.0% (3/20) had a decrease in the number of white blood cells (WBC) (<4 × 10^9^/L), and 2 had diarrhea and infusion, who reported dizziness, chest tightness, cold sweat, and rashes on the body. The symptoms disappeared within the next 2-3 days without any medical intervention. No other obviously adverse effects were reported, and the medication was not affected. Of the 22 patients in the conventional therapy group, 22.7% (5/22) had a decrease in WBC numbers (<4 × 10^9^/L).

## 4. Discussion

The pathogenesis and etiology of CD are not yet well understood so far. It may be caused by environmental factors, immune abnormalities, infection, susceptibility factors, diet structure, and genetic factors [9]. The incidence rate of CD with fistula incidence is 11%-12% [10, 11]. Treatment for CD complicated with intestinal fistula is very important. This prospective study aimed to provide clinical evidence for the application of EN in the treatment of CD complicated with intestinal fistula.

Previous studies have shown that, during IFX treatment, patients with a normal CRP level easily achieved a significant clinical response and had a better prognosis than patients with CRP >5 mg/L [12, 13]. At the 14th and the 30th week of follow-up and assessment of the disease, ESR and CRP levels declined compared with the level before treatment in the IFX treatment group. The differences were statistically significant (*P* < 0.01) in our study, suggesting that inflammation was temporally controlled.

As much as 86.4% of CD patients suffered from malnutrition [14–16]. Therefore, nutritional support is a key step for CD patients. In recent years, clinical studies have indicated that EN can be used as an immune modulator to maintain the treatment, which is beneficial to the long-term remission of CD. Its efficacy is equivalent to the immunosuppressive agent and without any adverse effects [17]. One of the important advantages of EN treatment is to induce remission together with simultaneous improvement of nutritional status of patients. Our results also confirmed that, after treatment for 14 weeks, BMI of patients in the IFX treatment group increased (*P* < 0.05). After 30 weeks of treatment, the BMI increased to 20.26 ± 2.65 kg/m^2^ which was statistically significant (*P* < 0.05). Moreover, the albumin levels of patients were significantly increased compared to that before IFX treatment, suggesting the nutritional status of the patients was also improved. In both groups, the increased BMI after treatment suggested that EN could correct malnutrition to some extent. In our study, peptison liquid, an amino acid predigested formula, was given to all the patients through nasogastric tube, which could provide proteins at a higher rate and reduces the burden on the gastrointestinal tract. The short peptides could stimulate intestinal mucosal epithelial growth, which in turn could promote the structural and functional recovery of the intestinal barrier. Importantly, the fewer residues and short peptide components do not act as antigens for the sensitive gut, and the absorption mode is close to a normal physiological protein absorption model.

In one of the DH' studies, Present et al. have showed in the first placebo-controlled trial that 68% of patients who received 5 mg/kg of infliximab and 56% of those who received 10 mg/kg achieved the primary end point, which was a reduction of 50% or more from baseline in the number of draining fistulas, as compared with 26% of the patients in the placebo group (*P* = 0.002 and *P* = 0.02, resp.). In addition, 55% and 38% of patients assigned to receive, respectively, 5 mg/kg and 10 mg/kg of infliximab had closure of all fistulas, as compared with 13% of the placebo group (*P* = 0.001 and *P* = 0.04, resp.) [18]. Hirai et al. [19] reported that the clinical remission rate of patients with CD who were treated with both EN and IFX was significantly higher than that in patients who did not receive EN. Notably, in our study, CDAI score and SES-CD decreased statistically significantly after treatment in the IFX treatment group. Compared to those in the conventional therapy group, the difference of CDAI and SES-CD between the two groups was significant (*P* < 0.05). Furthermore, fistula close was found in eight of 20 patients, including one anal fistula healing, in the IFX treatment group at 14 weeks. The cases of fistula healing were more than that in the conventional therapy group at 30 weeks (*P* < 0.05). Our study also showed that IFX combined with EN treatment could promote the healing of fistula (*n* = 18, 90%). Other researchers also supported that EN plays a supporting role in patients with malnutrition [20], and we confirmed that application of EN could improve the nutritional status of CD patients with intestinal fistulas and thus enhance the curative effects of IFX.

However, the limited cases were enrolled in this study, and the follow-up time was almost 4 years. More evidence was needed to conduct a large-scale study for a longer follow-up time in the future.

## 5. Conclusion

IFX in combination with EN induces and promotes remission in patients with CD complicated by fistula, with a better prospect of achieving a curative effect in the future. This treatment can also reduce inflammation of patients with CD, improve the nutritional status, and promote the healing of the fistula. It is worthy of clinical treatment.

## Figures and Tables

**Table 1:** The clinical characteristics of 42 patients with CD.

Clinical features	Conventional group (*n* = 22)	IFX group (*n* = 20)
Average age (year)	32.1 ± 11.8^*∗∗*^	31.6 ± 11.7
Sex (male, %)	13 (59%)^*∗∗*^	12 (60%)
Average CDAI score	309.9 ± 69.3^*∗∗*^	325.6 ± 70.8
*Duration of disease*		
<12 months	2 (9.1%)^*∗∗*^	2 (10.0%)
12–36 months	14 (63.6%)^*∗∗*^	13 (65.0%)
>36 months	6 (27.3%)^*∗∗*^	5 (25.0%)
*Lesion site*		
Terminal ileum (L1 type)	6 (27.3%)^*∗∗*^	6 (30.0%)
Colon (L2 type)	7 (31.8%)^*∗∗*^	6 (30.0%)
Ileum and colon (L3 type)	9 (40.9%)^*∗∗*^	8 (40.0%)
*Disease behavior*		
No stenosis or penetration (B1 type)	0 (0%)	0 (0%)
Stenosis (B2 type)	0 (0%)	0 (0%)
Penetration (B3 type)	22 (100%)^*∗∗*^	20 (100%)
*Stage of disease activity*		
Moderate active stage	14 (63.6%)^*∗∗*^	13 (65.0%)
Severe active stage	8 (36.4%)^*∗∗*^	7 (35.0%)
*Combined drug therapy*		
5-Aminosalicylic acid	22 (100%)^*∗∗*^	20 (100%)
Methylprednisolone	22 (100%)	0 (0%)
Imuran	22 (100%)	0 (0%)
*Associated with anal lesions*	16 (72.7%)^*∗∗*^	14 (70.0%)
*History of intestinal surgery*	5 (22.7%)^*∗∗*^	6 (30.0%)
*Extraintestinal manifestations*	4 (18.2%)^*∗∗*^	4 (20.0%)

Compared with the IFX treatment group: ^*∗∗*^ *P* > 0.05.

**Table 2:** Comparison of laboratory indexes and BMI before and after treatment in 42 cases.

Group	Treatment time	ESR (mm/h)	CRP (mg/L)	BMI (kg/m^2^)	ALB (g/L)
Conventional	Before treatment	31.78 ± 3.03	30.97 ± 6.86	17.66 ± 1.98	33.12 ± 1.61
	14 weeks after treatment	26.08 ± 2.89	10.87 ± 4.16	18.21 ± 2.09	37.88 ± 2.29
	30 weeks after treatment	14.24 ± 1.92^*∗∗*^	5.78 ± 2.59^*∗∗*^	19.98 ± 2.49^*∗∗*^	38.81 ± 2.69^*∗∗*^

IFX	Before treatment	36.43 ± 3.21	31.12 ± 6.99	17.52 ± 1.89	32.58 ± 1.67
	14 weeks after treatment	25.29 ± 2.92^*∗*^	12.35 ± 4.23^*∗*^	19.19 ± 2.13^*∗*^	38.92 ± 2.35^*∗*^
	30 weeks after treatment	13.21 ± 1.86^*∗*^	5.23 ± 2.63^*∗*^	20.26 ± 2.65^*∗*^	39.89 ± 2.72^*∗*^

Compared with that before treatment in the IFX treatment group: ^*∗*^ *P* < 0.05.

Compared with the IFX treatment group: ^*∗∗*^ *P* > 0.05.

**Table 3:** Comparison of CDAI, SES-CD, and fistula healing before and after treatment in all cases.

Group	Treatment time	CDAI	SES-CD	Fistula healing (cases)
Conventional	Before treatment	309.9 ± 69.3	8.1 ± 2.0	0 (0%)
	14 weeks after treatment	246.8 ± 63.9	6.2 ± 1.2	3 (13.6%)
	30 weeks after treatment	178.6 ± 46.6^▲▲^	4.9 ± 0.8^▲▲^	6 (27.3%)^▲▲^

IFX	Before treatment	325.6 ± 70.8	8.6 ± 2.2	0 (0%)
	14 weeks after treatment	235.5 ± 62.8^*∗*^	3.5 ± 0.8^*∗*^	8 (40.0%)
	30 weeks after treatment	125.6 ± 42.5^*∗*^	1.6 ± 0.5^*∗*^	18 (90.0%)

Compared with that before treatment in the IFX treatment group: ^*∗*^ *P* < 0.05.

Compared with the IFX treatment group: ^▲▲^ *P* < 0.05.

## References

[1] Bruining D. H., Loftus E. V. (2012). Crohn's disease in the emergency department: to CT or not CT. *Gastroenterology*.

[2] Neuman M. G., Nanau R. M. (2012). Single-nucleotide polymorphisms in inflammatory bowel disease. *Translational Research*.

[3] Randall C., Vizuete J., Wendorf G., Ayyar B., Constantine G. (2012). Current and emerging strategies in the management of Crohn's disease. *Best Practice and Research: Clinical Gastroenterology*.

[4] Ford A. C., Sandborn W. J., Khan K. J., Hanauer S. B., Talley N. J., Moayyedi P. (2011). Efficacy of biological therapies in inflammatory bowel disease: systematic review and meta-analysis. *The American Journal of Gastroenterology*.

[5] Group Ddcwbibds (2013). Inflammation in inflammatory bowel disease diagnosis and treatment of consensus (2012-Guangzhou). *Chinese Journal of Gastroenterology*.

[6] Sandborn W. J., Fazio V. W., Feagan B. G., Hanauer S. B. (2003). AGA technical review on perianal Crohn's disease. *Gastroenterology*.

[7] Daperno M., D'Haens G., Van Assche G. (2004). Development and validation of a new, simplified endoscopic activity score for Crohn's disease: the SES-CD. *Gastrointestinal Endoscopy*.

[8] Sands B. E., Anderson F. H., Bernstein C. N. (2004). Infliximab maintenance therapy for fistulizing Crohn's disease. *The New England Journal of Medicine*.

[9] Louis E., Mary J.-Y., Vernier-Massouille G. (2012). Maintenance of remission among patients with Crohn's disease on antimetabolite therapy after infliximab therapy is stopped. *Gastroenterology*.

[10] Ouyang Q., Tandon R., Goh K.-L., Ooi C. J., Ogata H., Fiocchi C. (2005). The emergence of inflammatory bowel disease in the Asian Pacific region. *Current Opinion in Gastroenterology*.

[11] Jiang L., Xia B., Li J. (2006). Retrospective survey of 452 patients with inflammatory bowel disease in Wuhan City, central China. *Inflammatory Bowel Diseases*.

[12] Vermeire S., Van Assche G., Rutgeerts P. (2006). Laboratory markers in IBD: useful, magic, or unnecessary toys?. *Gut*.

[13] Louis E., Vermeire S., Rutgeerts P. (2002). A positive response to infliximab in Crohn disease: association with a higher systemic inflammation before treatment but not with -308 TNF gene polymorphism. *Scandinavian Journal of Gastroenterology*.

[14] Massironi S., Rossi R. E., Cavalcoli F. A., Della Valle S., Fraquelli M., Conte D. (2013). Nutritional deficiencies in inflammatory bowel disease: therapeutic approaches. *Clinical Nutrition*.

[15] Hartman C., Eliakim R., Shamir R. (2009). Nutritional status and nutritional therapy in inflammatory bowel diseases. *World Journal of Gastroenterology*.

[16] Gong J. F., Niu L. Y., Wen-Kui Y. U., Zhu W. M., Ning L. I., Jie-Shou L. I. (2009). *Perioperative Nutrition Support in Patients with Crohn's Disease*.

[17] Hanai H., Iida T., Takeuchi K. (2012). Nutritional therapy versus 6-mercaptopurine as maintenance therapy in patients with Crohn's disease. *Digestive and Liver Disease*.

[18] Present D. H., Rutgeerts P., Targan S. (1999). Infliximab for the treatment of fistulas in patients with Crohn's disease. *The New England Journal of Medicine*.

[19] Hirai F., Ishihara H., Yada S. (2013). Effectiveness of concomitant enteral nutrition therapy and infliximab for maintenance treatment of crohn's disease in adults. *Digestive Diseases and Sciences*.

[20] Yamamoto T., Nakahigashi M., Umegae S., Matsumoto K. (2010). Enteral nutrition for the maintenance of remission in Crohn's disease: a systematic review. *European Journal of Gastroenterology & Hepatology*.

